# Item difficulty index, discrimination index, and reliability of the 26 health professions licensing examinations in 2024, Korea: a psychometric study

**DOI:** 10.3352/jeehp.2025.22.38

**Published:** 2025-12-15

**Authors:** Yoon Hee Kim, Bo Hyun Kim, Joonki Kim, Bokyoung Jung, Sangyoung Bae

**Affiliations:** Research and Development Division, Korea Health Personnel Licensing Examination Institute, Seoul, Korea; Hallym University, Korea

The Korea Health Personnel Licensing Examination Institute administers national licensing examinations annually to determine whether candidates meet the essential competencies required for healthcare practice. To ensure that these examinations remain valid and appropriate, post-exam analyses of item difficulty, discrimination, and reliability are routinely conducted. Questions with extremely high or low difficulty or discrimination scores may require review and revision, offering meaningful insights for improving future tests. This study evaluated the item difficulty, discrimination, and reliability of 26 health personnel licensing exams held in Korea in 2024 to assess their overall suitability. It also explored the relationship between the average item difficulty and average item discrimination indices. This work follows a previous report covering the 2022 and 2023 licensing exams [1,2].

Since this study involved analyzing test results rather than human subjects, institutional review board approval and informed consent were not required.

Item analysis was performed on the 26 examinations using classical test theory. Item difficulty (P) was defined as the proportion of examinees who answered an item correctly, ranging from 0 to 1. Item discrimination was measured using the upper and lower 27% method, which compares the difference in difficulty between the top and bottom 27% of examinees, as well as the correlation of individual item scores with total test scores. Discrimination indices and reliability were calculated only for exams with more than 100 participants. Statistical analyses were conducted using IBM SPSS ver. 21.0 (IBM Corp.).

Pass rates across the 26 examinations varied widely, ranging from 25.0% to 100%. The number of examinees per exam ranged from 12 (for health educators–level 1 and midwives) to 181,890 (for care workers). Exams for physicians, dentists, midwives, nurses, doctors of Korean medicine, and pharmacists all had pass rates exceeding 90%. Notably, the midwives’ exam had a 100% pass rate, although it included only 12 candidates (Table 1).

Table 2 summarizes the results of the item analysis for the 2024 examinations. All exams showed high reliability, with the lowest reliability observed for the emergency medical technicians–level 2 exam (0.880). The average difficulty indices ranged from 53.1% (health educators–level 2) to 80.1% (physicians), while the average item-total correlations ranged from 0.19 (doctors of Korean medicine) to 0.43 (opticians).

According to classical test theory, average difficulty indices are classified as moderate (50%–60%), somewhat easy (60%–70%), easy (70%–80%), and very easy (80% or above). Based on these categories, the 2024 exams’ difficulty indices were rated as very easy for the occupational therapists and physicians exams, easy for 14 exams, and somewhat easy for 13 exams. For medical professions such as physicians (80.1%), dentists (77.3%), nurses (79.5%), and doctors of Korean medicine (74.6%), the difficulty indices fell into the very easy or easy categories, with corresponding pass rates exceeding 90%.

In classical test theory, discrimination indices below 0.30 are generally considered low. Seventeen examinations demonstrated low discrimination, whereas 11 showed good discrimination. For example, the doctors of Korean medicine exam had a relatively low discrimination index (average item-total correlation of 0.19), while the opticians exam demonstrated good discrimination (average item-total correlation of 0.41). All tests maintained high reliability, with Cronbach’s alpha values of 0.880 or higher, ensuring consistent measurement across examinations.

In 2022, the national licensing examinations had 468,352 candidates with an 89.4% pass rate. In 2023, there were 446,054 candidates with an 87.0% pass rate. In 2024, both the number of candidates (286,922) and the number of successful examinees (244,205) decreased, resulting in an 85.1% pass rate. The pass rate range shifted from 52.2%–100.0% in 2023 to 25.0%–100.0% in 2024. Analysis of difficulty indices for both years showed that most exams remained relatively easy. Twenty examinations in 2024 showed decreased difficulty compared to 2023, mainly shifting among the easy, very easy, and somewhat easy categories, without major overall changes. Additionally, the Care Workers Qualification Examination transitioned to computer-based testing in 2023, leading to non-disclosure of test items and analysis results, which limited direct comparisons.

In 2024, 17 examinations had lower discrimination indices than in 2023, and 12 showed decreases in item-total correlation coefficients. Despite these declines, the overall discrimination indices for 2024 did not differ significantly from those in 2023, suggesting no substantial reduction in discriminative capacity. Confidentiality policies restrict the release of detailed item analyses and test results, limiting comparisons with previous years and constraining broader academic discussion of these examinations.

In summary, Korea’s 2024 national health personnel licensing examinations exhibited satisfactory levels of difficulty, discrimination, and reliability. Nonetheless, 5 of the 26 exams had low discrimination indices despite having appropriate difficulty levels.

## Figures and Tables

**Table 1.** Pass rates for examinees in the 26 national health professions licensing examinations in Korea, 2024

Job titles	No. of examinees	No. of successful candidates	Pass rate (%)
Assistive technology professionals (6th)	349	290	83.1
Care workers^a)^	181,890	156,081	85.8
Dental hygienists (52nd)	4,837	4,182	86.5
Dental technicians (52nd)	910	762	83.7
Dentists (77th)	778	721	93.3
Dietitians (48th)	5,065	3,538	69.9
Emergency medical technicians–level 1 (30th)	1,629	1,206	74.0
Emergency medical technicians–level 2 (30th)	1,172	989	84.4
Health educators–level 1 (15th)	12	3	25.0
Health educators–level 2 (15th)	135	77	57.0
Health educators–level 3 (15th)	884	638	72.2
Health information managers (41st)	2,268	1,360	60.0
Oriental medicine pharmacists (24th)	146	128	87.7
Medical technologists (52nd)	2,975	2,489	83.7
Midwives (35th)	12	12	100.0
Nurses (64th)	24,377	23,567	96.7
Nursing aides (1st half)	16,370	14,830	90.6
Nursing aides (2nd half)	15,428	13,639	88.4
Occupational therapists (52nd)	1,774	1,591	89.7
Opticians (37th)	1,466	1,043	71.1
Doctors of Korean medicine (79th)	797	773	97.0
Oriental medicine dispensers (31st)	-	-	
Pharmacists (75th)	2,071	1,879	90.7
Physical therapists (52nd)	5,176	3,974	76.8
Physicians (88th)	3,231	3,045	94.2
Prosthetists and orthotists (25th)	123	85	69.1
Radiological technologists (52nd)	2,787	2,181	78.3
Rehabilitation counselors–level 1 (8th)	488	361	74.0
Rehabilitation counselors–level 2 (8th)	119	99	83.2
Sanitary technicians (46th)	7,610	3,514	46.2
Speech-language pathologists–level 1 (13th)	987	507	51.4
Speech-language pathologists–level 2 (13th)	1,056	641	60.7

a) In 2023, the Care Workers Qualification Examination transitioned to a computer-based test with continuous availability. In 2024, paper-based testing was not conducted.

**Table 2.** Difficulty index, upper and lower group discrimination index, item-total correlation, and reliability index presented as Cronbach α for 26 health professional licensing examinations in Korea, 2024

Job title	No. of subjects	No. of items	Difficulty index (%)	Upper and lower group discrimination^a)^	Item-total correlation^a)^	Reliability (Cronbach α)
Assistive technology professionals (6th)	2	170	66.8±26.6	0.20±0.14	0.22±0.11	0.880
Care workers^b)^	2	80				
Dental hygienists (52nd)	2	200	74.3±18.1	0.26±0.12	0.28±0.10	0.945
Dental technicians (52nd)	3	205	73.9±18.5	0.31±0.15	0.33±0.12	0.962
Dentists (76th)	13	321	77.3±22.5	0.20±0.14	0.22±0.10	0.944
Dietitians (48th)	4	220	66.7±21.1	0.33±0.17	0.30±0.13	0.959
Emergency medical technicians–level 1 (30th)	5	230	67.0±21.0	0.27±0.14	0.25±0.11	0.941
Emergency medical technicians–level 2 (30th)	5	140	69.3±20.6	0.22±0.13	0.21±0.10	0.880
Health educators–level 1 (15th)^a)^	3	60	53.1±29.4	-	-	-
Health educators–level 2 (15th)	8	180	61.9±20.2	0.27±0.19	0.27±0.15	0.925
Health educators–level 3 (15th)	4	110	66.6±22.4	0.28±0.17	0.27±0.14	0.889
Health information managers (41st)	3	230	65.5±20.7	0.35±0.17	0.32±0.12	0.964
Medical technologists (52nd)	4	280	75.7±16.5	0.34±0.13	0.37±0.11	0.977
Midwives (35th)^a)^	4	165	77.7±23.8	-	-	-
Nurses (64th)	8	295	79.5±20.1	0.18±0.12	0.23±0.09	0.936
Nursing aides (1st half)	4	100	79.7±19.2	0.30±0.18	0.36±0.12	0.932
Nursing aides (2nd half)	4	100	78.4±16.7	0.31±0.15	0.37±0.11	0.932
Occupational therapists (52nd)	4	240	80.0±16.2	0.27±0.15	0.32±0.11	0.964
Opticians (37th)	4	250	70.8±16.3	0.43±0.17	0.41±0.14	0.981
Doctors of Korean medicine (79th)	11	340	74.6±23.0	0.16±0.12	0.19±0.10	0.925
Oriental medicine pharmacists (25th)	3	250	73.0±23.4	0.24±0.15	0.31±0.17	0.958
Pharmacists (75th)	4	350	71.2±22.4	0.21±0.11	0.25±0.11	0.953
Physical therapists (52nd)	5	260	69.5±20.4	0.27±0.17	0.28±0.12	0.955
Physicians (88th)	3	320	80.1±20.1	0.18±0.13	0.23±0.11	0.942
Prosthetists and orthotists (25th)	7	210	65.0±21.4	0.30±0.19	0.26±0.15	0.945
Radiological technologists (52nd)	4	250	73.5±16.9	0.36±0.16	0.36±0.13	0.975
Rehabilitation counselors–level 1 (8th)	7	150	67.5±23.2	0.24±0.14	0.25±0.11	0.896
Rehabilitation counselors–level 2 (8th)	7	120	73.6±19.2	0.26±0.17	0.27±0.14	0.892
Sanitary technicians (46th)	6	220	61.7±20.9	0.31±0.17	0.28±0.14	0.950
Speech-language pathologists–level 1 (13th)	6	140	60.9±22.9	0.28±0.16	0.26±0.13	0.903
Speech-language pathologists–level 2 (13th)	5	150	63.3±19.9	0.36±0.15	0.33±0.12	0.947

Values are presented as number or mean±standard deviation unless otherwise stated.

^a)^Discrimination indices and reliability were not calculated for examinations with fewer than 100 candidates. ^b)^The item analysis results for the Care Workers Qualification Examination were not disclosed.
